# MicroRNA-34a-5p: A pivotal therapeutic target in gallbladder cancer

**DOI:** 10.1016/j.omton.2024.200765

**Published:** 2024-02-08

**Authors:** Takashi Oda, Koichiro Tsutsumi, Taisuke Obata, Eijiro Ueta, Tatsuya Kikuchi, Soichiro Ako, Yuki Fujii, Tatsuhiro Yamazaki, Daisuke Uchida, Kazuyuki Matsumoto, Shigeru Horiguchi, Hironari Kato, Hiroyuki Okada, Ryota Chijimatsu, Motoyuki Otsuka

**Affiliations:** 1 Department of Gastroenterology and Hepatology, Okayama University Graduate School of Medicine, Dentistry and Pharmaceutical Science, Okayama, Japan; 2 Department of Gastroenterology, Okayama University Hospital, Okayama, Japan; 3 Center for Comprehensive Genomic Medicine, Okayama University Hospital, Okayama, Japan

**Keywords:** MT: Regular Issue, carcinogenesis, organoid, gallbladder cancer, microRNA, miR-34a-5p, cell cycle, CDK6, xenograft, biliary tract, therapy

## Abstract

Gallbladder cancer incidence has been increasing globally, and it remains challenging to expect long prognosis with the current systemic chemotherapy. We identified a novel nucleic acid-mediated therapeutic target against gallbladder cancer by using innovative organoid-based gallbladder cancer models generated from *Kras*^LSL-G12D/+^; *Trp53*^f/f^ mice. Using comprehensive microRNA expression analyses and a bioinformatics approach, we identified significant microRNA-34a-5p downregulation in both murine gallbladder cancer organoids and resected human gallbladder cancer specimens. In three different human gallbladder cancer cell lines, forced microRNA-34a-5p expression inhibited cell proliferation and induced cell-cycle arrest at the G1 phase by suppressing direct target (*CDK6*) expression. Furthermore, comprehensive RNA sequencing revealed the significant enrichment of gene sets related to the cell-cycle regulators after microRNA-34a-5p expression in gallbladder cancer cells. In a murine xenograft model, locally injected microRNA-34a-5p mimics significantly inhibited gallbladder cancer progression and downregulated *CDK6* expression. These results provide a rationale for promising therapeutics against gallbladder cancer by microRNA-34a-5p injection, as well as a strategy to explore therapeutic targets against cancers using organoid-based models, especially for those lacking useful genetically engineered murine models, such as gallbladder cancer.

## Graphical abstract

**Figure:**
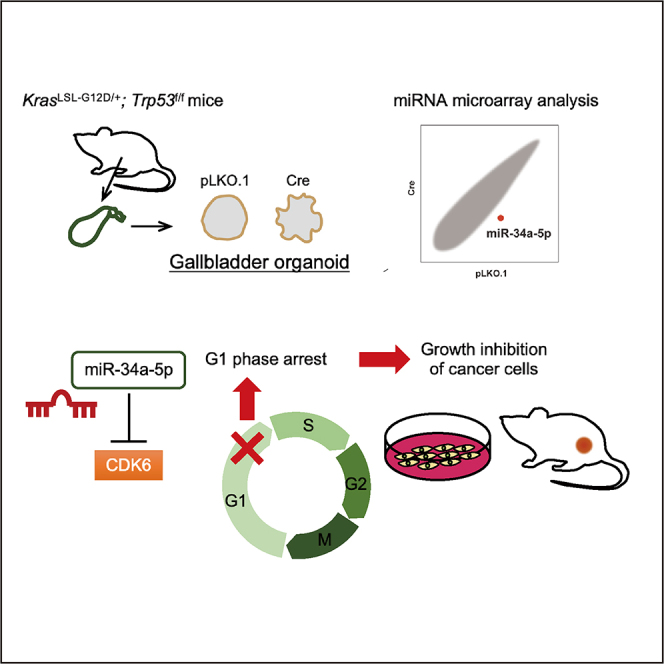

## Teaser

Oda and colleagues identified significant microRNA-34a-5p downregulation in both murine gallbladder cancer (GBC) organoids and resected human GBC specimens by using innovative organoid-based GBC models generated from *Kras*^LSL-G12D/+^; *Trp53*^f/f^ mice. miR-34a-5p forced expression leads to efficient GBC progression suppression via cell-cycle inhibition by CDK6 downregulation.

## Introduction

The incidence of gallbladder cancer (GBC), a type of biliary tract cancer (BTC), has increased worldwide. However, because it is difficult to diagnose at an early stage due to the lack of clinical manifestations,^1^ radical surgery is rarely performed. Moreover, although systemic chemotherapy has progressed, it is not sufficient to improve prognosis, and the 5-year survival rate remains <20%.^2^ Recently, whole-exome sequencing has revealed a landscape of genomic alterations in BTC, and various altered genes, such as *TP53*, *KRAS*, *SMAD4*, *ARID1A*, and *PIK3CA*, have been identified in GBC.^3^^,^^4^ In some cases with *NTRK* gene fusion-positive tumors, mismatch repair-deficient tumors, or microsatellite instability-high tumors, *NTRK* inhibitors or immune checkpoint inhibitors are expected to exhibit a high treatment effect^5^^,^^6^; however, patients with such gene alterations account for <10% of patients with BTC. Therefore, there is an urgent need to develop novel treatments, including molecular-targeted therapies, for patients with GBC.

To date, several studies have been conducted to investigate therapeutic targets using surgically resected specimens from patients with GBC.^4^^,^^7^^,^^8^^,^^9^^,^^10^ However, only a few targets applicable to clinical use have been identified. This limited success can be attributed to numerous factors such as the analysis of bulk samples, a high degree of intra- and intertumor heterogeneity, and the use of unsuitable control samples. In addition, the lack of an ideal genetically engineered murine model that accurately reflects human primary GBC has hampered research. In recent years, three-dimensional (3D) cultured organoid-based research has become widespread in cancer biology.^11^ This advanced *in vitro* culture tool allows for long-term culture of normal stem cells under physiological conditions^12^ and provides a high-throughput drug screening platform with improved reliability compared to conventional 2D culture systems.^13^ Regarding the gallbladder (GB), an organoid-based carcinogenesis model using GB organoids derived from *Kras*^LSL-G12D/+^; *Trp53*^f/f^ mice generated murine primary GBC organoids with mutant Kras and Trp53 loss.^14^^,^^15^^,^^16^ These organoid-based carcinogenesis models present considerable potential as innovative tools for exploring GBC biology.

With recent advances in nucleic acid-mediated therapies, novel therapeutic strategies targeting microRNAs (miRNAs) have been developed for various types of cancers,^17^ including GBC.^18^^,^^19^^,^^20^ However, the crucial miRNAs associated with GBC progression that could serve as high-priority therapeutic targets have not yet been identified.

In this study, we comprehensively examined the miRNA expression profiles of *Kras*-activated and *Trp53-*deleted tumorigenic GB organoids mimicking GBC and normal GB organoids mimicking the normal GB. We compared these profiles and identified pivotal therapeutic miRNA targets that regulate GBC progression. A direct gene target and comprehensive transcriptional changes after forced miRNA expression were determined, and the effects of local administration of the miRNA into mouse xenograft models were evaluated to develop a new therapeutic option against GBC.

## Results

### miR-34a-5p is downregulated in GBC

**Figure 1:**
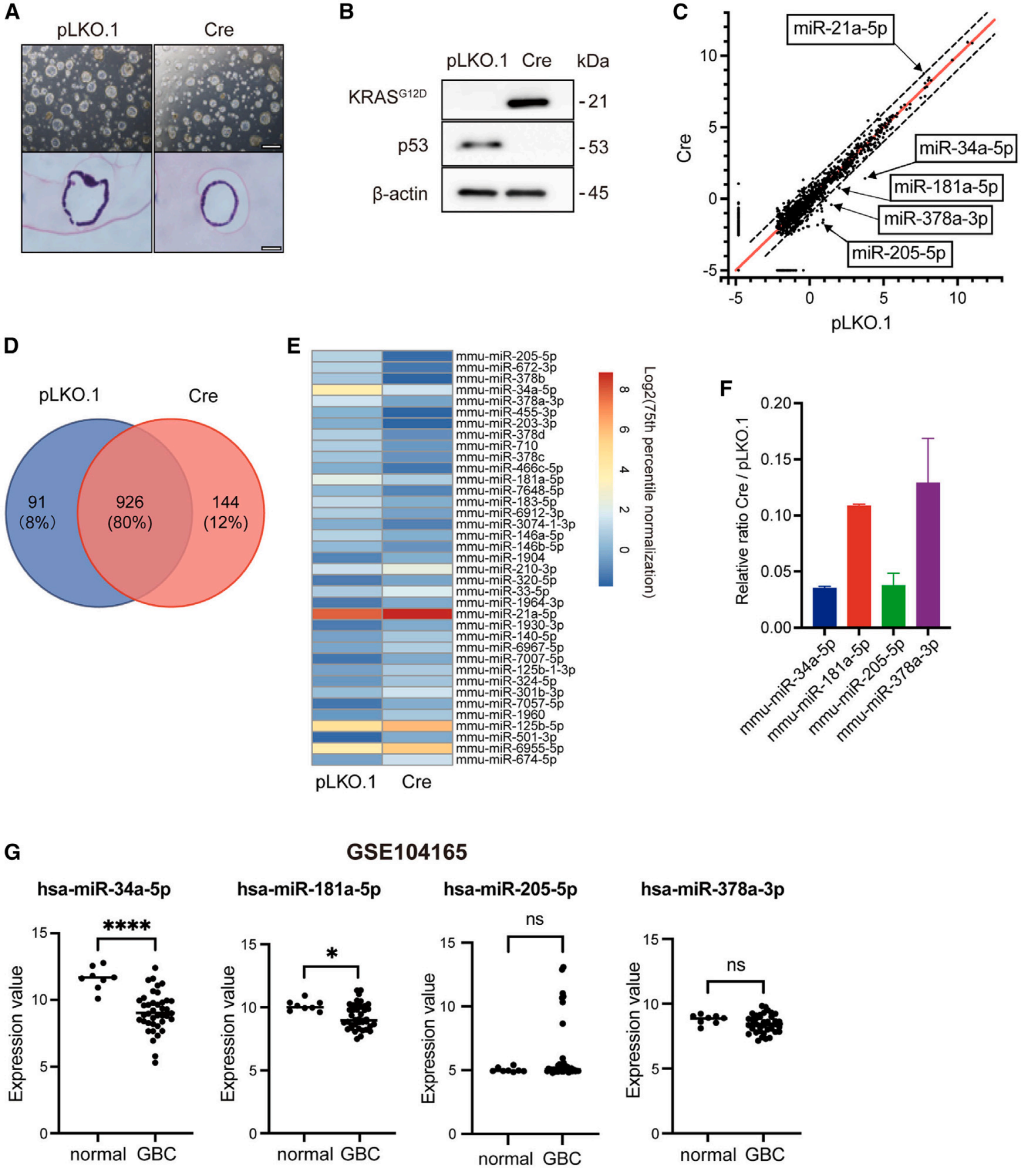
Differentially expressed miRNA identification in GBC-mimicking organoids (A) Phase-contrast images (top) and H&E (HE) staining (bottom) of pLKO.1-transduced control organoids (normal GB organoids) and GBC organoids (Cre-transduced organoids) derived from *Kras*^LSL-G12D/+^; *Trp53*^f/f^ mice. Scale bars indicate 500 μm (top) and 50 μm (bottom). (B) Western blotting confirmed the Cre-mediated induction of Kras^G12D^ and deletion of Trp53 in GBC organoids, with β-actin serving as a loading control. (C) miRNA expression profiles in normal GB organoids and GBC organoids using miRNA microarrays. (D) Venn diagram showing the number of miRNAs exclusively or commonly detected in normal GB organoids (pLKO.1) and GBC organoids (Cre). (E) Heatmap depicts 18 miRNAs with higher expression levels (log_2_FC > 1) and 19 miRNAs with lower expression levels (log_2_FC < −1) in GBC organoids compared with normal GB organoids. (F) The decreased expression levels of 4 miRNAs (miR-34a-5p, miR-181a-5p, miR-378a-3p, and miR-205-5p) in GBC organoids were confirmed by qRT-PCR. The miRNA expression levels were normalized to snoRNA202 expression levels. Data are presented as the mean ± SD (n = 3). (G) The expression levels of miR-34a-5p showed the most significant decrease in human GBC tissues compared to those in normal GB tissues (log_2_FC = −2.50) in GSE104165. The horizontal lines indicate the median values. ∗p *<* 0.05; ∗∗∗∗p *<* 0.0001.

To identify miRNAs that were differentially expressed between normal GB and GBC, we conducted a comprehensive miRNA expression analysis by comparing organoids mimicking normal GB and GBC. Although both types of organoids exhibited cyst formation (Figure 1A), Cre-mediated induction of *Kras*^G12D^ and deletion of *Trp53* were observed only in GBC organoids (Figures 1B and S6). Using qualified RNAs extracted from these organoids (Figure S1), the study revealed that the expression levels of 144 kinds of miRNAs and 91 miRNAs were higher (log_2_FC [fold change] > 1) and lower (log_2_FC < −1), respectively, in GBC organoids than in normal GB organoids in the miRNA microarray analyses (Figures 1C and 1D). When focusing on miRNAs with a signal intensity >25, the expression levels of 18 miRNAs were higher (log_2_FC > 1) and those of 19 miRNAs were lower (log_2_FC < −1) in GBC organoids than in normal GB organoids. Although miR-21a-5p, a well-known oncogenic miRNA, was upregulated, four types of miRNAs—miR-34a-5p, miR-181a-5p, miR-378a-3p, and miR-205-5p, which have been implicated as tumor suppressors in various cancers^21^^,^^22^^,^^23^^,^^24^^,^^25^—exhibited decreased expression levels in GBC organoids (Figure 1E). Through validation of the expression levels of these four miRNAs by quantitative reverse transcriptase-polymerase chain reaction (qRT-PCR), the downregulation of miR-34a-5p expression was confirmed, with the largest differences between normal GB and GBC organoids (Figure 1F). Similarly, in a public miRNA microarray database using human resected specimens (GSE104165),^26^ miR-34a-5p expression levels showed the most significant decrease in GBC tissues compared to normal GB tissues (Figure 1G). Based on these findings, we hypothesized that miR-34a-5p plays a pivotal role in GBC and could serve as a promising candidate for miRNA-based targeted therapy for GBC.

### Forced miR-34a-5p expression inhibits cell proliferation and viability in GBC cell lines

**Figure 2:**
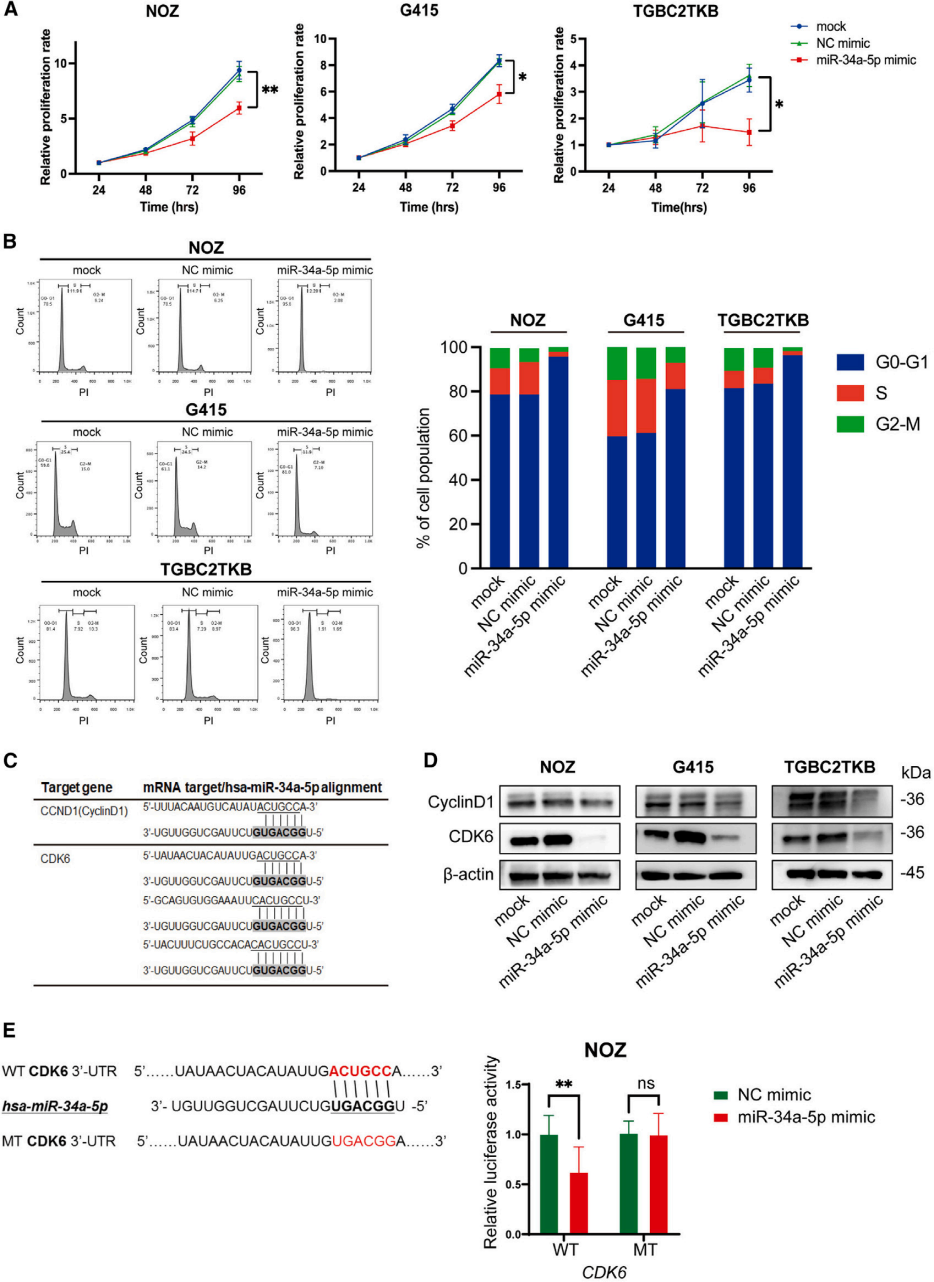
miR-34a-5p inhibits the cell proliferation of GBC cells in 2D cell cultures (A) Relative proliferation rate in human GBC cell lines after transfection with miR-34a-5p mimics was significantly suppressed compared to that in the negative control using an MTT assay. Data are presented as the means ± SDs (n *=* 3). ∗p *<* 0.05; ∗∗p *<* 0.01. (B) Cell-cycle arrest at the G1 phase was induced in GBC cell lines after transfection with miR-34a-5p mimics by flow cytometry. Representative images from 3 independent experiments are shown. (C) Potential target sequences in the 3′ UTR of cyclin D1 and CDK6 are shown. Target sequences (top sequences) and miR-34a-5p sequences (bottom sequences) were aligned with the highlighted complementary sequences at the seed sequences in miR-34a-5p using underlined text and bold font. (D) Western blot showing a reduction in cyclin D1 and CDK6 expression levels in GBC cell lines after transfection with miR-34a-5p mimics compared to the negative control. β-Actin was used as a loading control. Representative images from 3 independent experiments are shown. (E) The luciferase reporter constructs conjugated with the WT 3′ UTR sequences of *CDK6* or 3′ UTR sequences with MTs at possible target sequences were used. Sequences complementary to the seed sequences are indicated in red. miR-34a-5p significantly decreased the relative luciferase activity of the construct with the WT 3′ UTR sequences of *CDK6* in NOZ cells. Data from triplicate experiments are expressed as the mean ± SD. ∗∗p *<* 0.01.

Three types of human GBC cell lines (G415, NOZ, and TGBC2TKB) were used for subsequent *in vitro* studies. To explore the potential of miR-34a-5p as a therapeutic target for GBC, these cell lines were transfected with an miR-34a-5p mimic. Significant inhibition of cell proliferation in cells transfected with the mimic was observed compared to that in the negative control in 2D cell cultures (NOZ, p < 0.01; G415 and TGBC2TKB, p *<* 0.05; Figure 2A) in a dose-dependent manner (Figure S2). These results suggest that forced miR-34a-5p expression can decrease the proliferation rate and viability of GBC cells.

### Forced miR-34a-5p expression induces cell-cycle arrest at the G1 phase in GBC cell lines

Mechanisms underlying these phenomena were determined by assessing the cell cycle. The miR-34a-5p mimic expression induced a remarkable increase in the percentage of cells in the G1 phase in all cell lines (Figure 2B). According to the miRDB database, *CDK6*, an important kinase for the G1/S transition, and *CCND1*, which forms a complex with and functions as a regulatory subunit of CDK6,^27^ were identified as direct targets of miR-34a-5p (target scores: 92 and 58, respectively) (Figure 2C). The protein expression levels of CDK6 and cyclin D1 were substantially decreased in all three cell lines by forced expression of the miR-34a-5p mimic (Figures 2D and S6). These findings suggest that overexpression of miR-34a-5p induces cell-cycle arrest at the G1 phase, at least partly by downregulating the expression of CDK6 and cyclin D1, leading to cell proliferation inhibition in GBC cell lines.

### CDK6 is a direct target of miR-34a-5p

To further explore whether the 3′ UTR of *CDK6* is a direct target of miR-34a-5p, luciferase-based reporters were used (Figure 2E). Cotransfection of NOZ cells with the reporter construct containing the wild-type (WT) or mutant-type (MT) *CDK6* 3′ UTR sequences at the downstream of the luciferase gene and the miR-34a-5p mimic resulted in a significant decrease in luciferase activity compared to cells transfected with the negative control mimic, only when using the reporter construct with WT *CDK6* 3′ UTR sequences (p < 0.01; Figure 2E). These results, together with the marked decrease in CDK6 expression in GBC cells transfected with the miR-34a-5p mimic (Figure 2D), indicated that miR-34a-5p predominantly inhibits the cell cycle and cell proliferation, partially via direct CDK6 downregulation in GBC cells.

### Differentially expressed gene (DEG) enrichment related to cell cycle by miR-34a-5p expression in GBC cells

**Figure 3:**
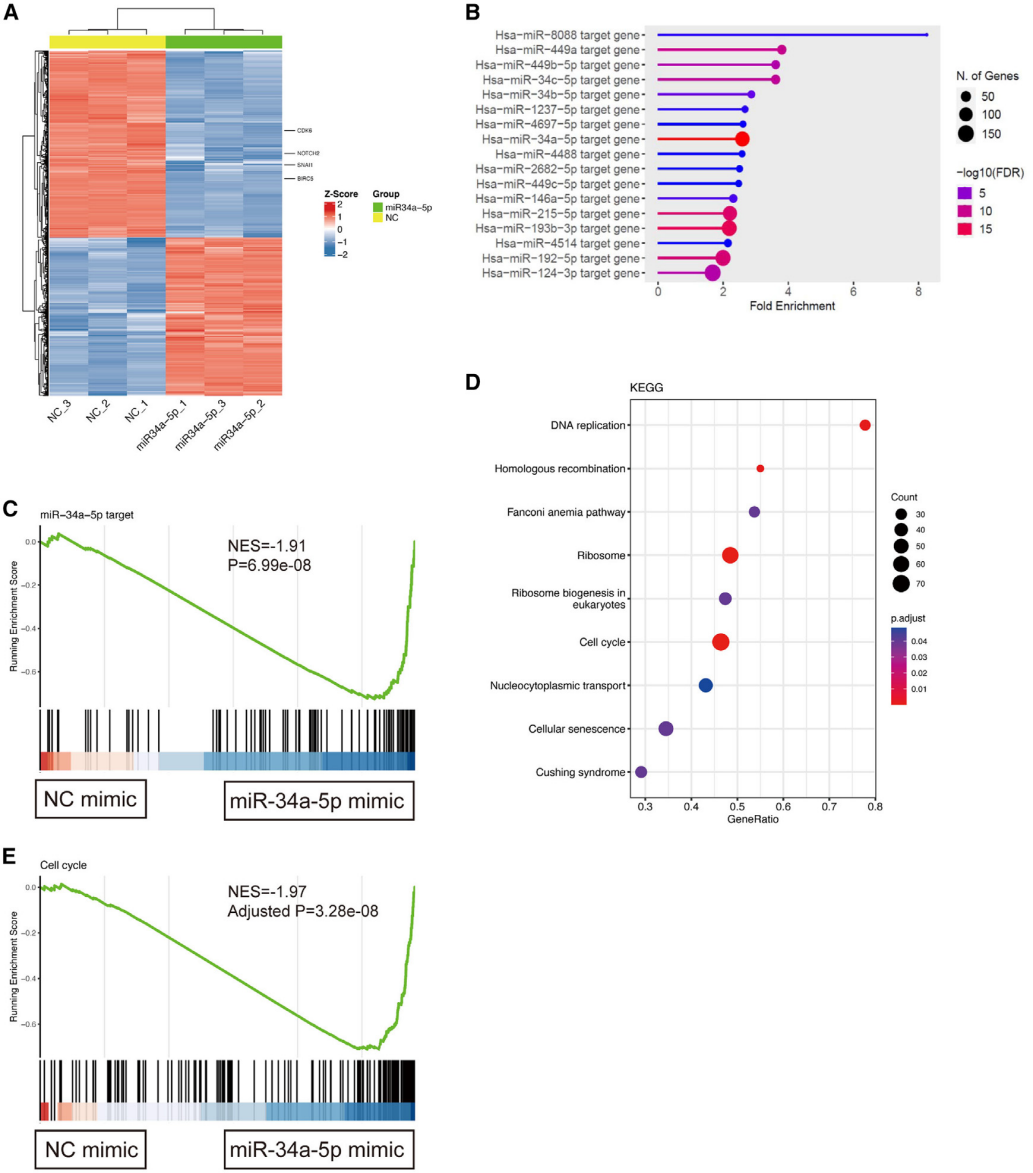
Enrichment of gene sets related to cell-cycle regulation in the miR-34a-5p mimic-transfected GBC cells (A) Heatmap showing hierarchical clustering based on DEG expression in miR-34a-5p-transfected cells (n = 3) and controls (n = 3). DEGs were identified with an FDR < 0.01 and an absolute FC >2. (B) The downregulated genes exhibited significant enrichment in the miR-34a-5p target gene set, as evidenced by enrichment analyses (FDR = 9.7e−20). The most enriched miRNA target genes along with their FDR and gene counts are listed. (C) GSEA plot demonstrating the enrichment of miR-34a-5p target gene sets in miR-34a-5p-transfected GBC cells compared to the negative control (p = 6.99e−08). (D) GSEA results demonstrate significant enrichment of gene sets related to the cell cycle following the miR-34a-5p-transfection. The most enriched biological processes, along with their p values and gene counts, are presented. (E) GSEA plot demonstrating the enrichment of cell-cycle-related gene sets in miR-34a-5p-transfected GBC cells compared to the negative control (adjusted p = 3.28e−08).

To determine the transcriptomic differences between control and miR-34a-5p mimic-transfected GBC cell lines, RNA samples from paired NOZ cells were used for RNA sequencing (RNA-seq) analyses. The principal-component analysis plot showed distinct clustering between NOZ cells transfected with miR-34a-5p and negative controls (Figure S3). Compared to the control, miR-34a-5p-transfected NOZ cells exhibited 2,437 significant DEGs, with 927 upregulated and 1,037 downregulated genes (Figure 3A). As expected, the downregulated genes showed the most significant association with the miR-34a-5p target genes, as determined by enrichment analysis and gene set enrichment analysis (GSEA) (Figure 3B, false discovery rate [FDR] = 9.7e−20; Figure 3C, p = 6.99e−08). Known miR-34a-5p target gene expressions such as *Snail1* (target score: 94), *BIRC5* (score not shown),^28^ *Notch2* (target score: 84) and *CDK6* were significantly decreased (Figure S4). Furthermore, Kyoto Encyclopedia of Genes and Genomes (KEGG) analysis of these genes revealed a significant enrichment of biological processes related to the inhibition of the cell cycle and G1-to-S-phase transition (Figures 3D and 3E, adjusted p = 3.28e−08; Figure S5), which is consistent with the aforementioned results showing the inhibition of cell-cycle progression and proliferation in miR-34a-5p-transfected GBC cell lines.

### miR-34a-5p suppresses GBC growth in the murine xenograft model

**Figure 4:**
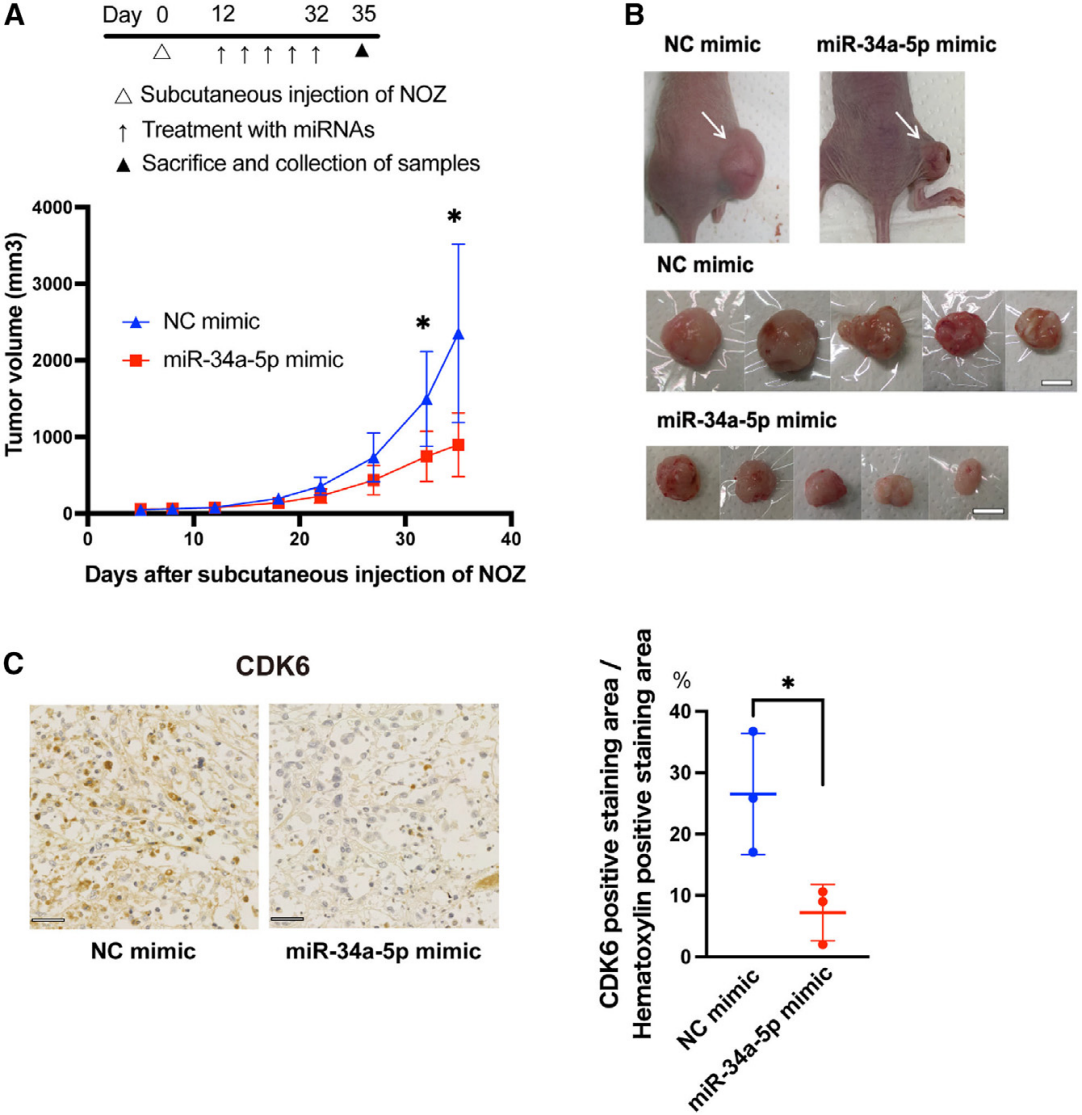
Local miR-34a-5p administration suppresses GBC growth *in vivo* (A) Subcutaneous miR-34a-5p mimic or negative control with atelocollagen injection around the NOZ tumor was repeated every 4 or 5 days, for a total of 5 sessions. Error bars indicate the mean ± SD; n = 5 mice per group. (B) miR-34a-5p mimics significantly suppressed tumor growth compared to the negative control. Macroscopic images of tumors from each group after 5 injections over 21 days are shown. The scale bar indicates 10 mm. (C) IHC staining of CDK6 protein expression (brown in the nucleus) in harvested subcutaneous tumors after 5 injections for 21 days (left). The scale bar indicates 50 μm. The injection of miR-34a-5p mimics significantly decreased the expression levels of CDK6 protein. Error bars represent the mean ± SD (n = 3 in each group) (right). ∗p *<* 0.05.

To confirm the suppressive function of miR-34a-5p in GBC *in vivo*, we used a GBC xenograft model. When the average size of the subcutaneously implanted tumors reached ∼80 mm^3^, a mixture of miR-34a-5p or a negative control with atelocollagen was injected around the tumors every 4–5 days for 3 weeks (Figure 4A). Compared to the negative controls, miR-34a-5p significantly inhibited GBC tumor growth after five injections (Figures 4A and 4B). miR-34a-5p injection significantly decreased the CDK6 expression levels in the tumors compared to those in the negative control (Figure 4C), suggesting that forced expression of miR-34a-5p by direct injection also has the potential to suppress GBC growth *in vivo*.

## Discussion

Because only a small number of therapeutic options are currently available for GBC, novel therapeutic development is urgently required. In this study, we uncovered the vital role of miR-34a-5p in GBC and determined that miR-34a-5p forced expression leads to efficient GBC progression suppression via cell-cycle inhibition, at least partly by *CDK6* downregulation, a direct target of this miRNA.

The 3D organoid culture system has been established as a physiological model that closely mimics the structure and differentiation that occur in the body compared to 2D culture systems. In particular, these organoid models have enabled us to culture primary normal epithelial cells for a long time, including normal GB cells, and have therefore led us to precisely compare GBC and normal GB *in vitro*. Owing to the lack of genetically modified murine models for GBC presently, normal GB organoids and GBC organoids induced by *Kras* activation and *Trp53* loss with Cre expression used here present attractive alternatives for GBC studies. Although the induction of an oncogene and/or the regulation of a tumor suppressor gene in normal epithelial cells does not necessarily lead to tumorigenicity,^16^ it has already been confirmed that Cre-induced GBC organoids were actually tumorigenic when inoculated subcutaneously into mice, but normal GB organoids were not tumorigenic.^16^ Therefore, these organoids present promising tools for assessing the biology of GBC.

In this study, several miRNAs were found to be differentially expressed in GBC, including miR-34a-5p. Although miR-34a-5p is transcriptionally activated by *TP53*,^29^^,^^30^ the miR-34a expression is also regulated by other mechanisms, such as CpG methylation of its promoter region,^31^ sponge effects of long noncoding RNA,^32^^,^^33^ and chromosomal deletion.^24^ Therefore, its expression is likely not solely dependent on p53 status but also on other factors. *TP53* mutations are identified in over 40%–60% of GBC cases, establishing it as one of the predominant driver mutations for GBC.^3^^,^^34^ In addition, significantly decreased expression levels of miR-34a-5p were observed in most GBC tissues, as deduced from the results deposited in public databases, and the overexpression of miR-34a-5p resulted in growth inhibition in GBC cell lines, even those with WT *TP53*, such as G415 cells. Further examination using GB and GBC organoids with WT p53 may be necessary to determine the biological significance of miR-34a-5p in GBC in such cases.

miR-34a-5p is downregulated in a wide range of solid tumors and hematological malignancies.^25^ Specifically, miR-34a-5p directly regulates several target mRNAs encoding proteins related to cell-cycle transition (*CCND1*, *CDK6*, *Notch1*, and *Notch2*),^27^^,^^35^ apoptosis (*Bcl-2* and *BIRC5*),^36^^,^^37^^,^^38^^,^^39^ migration, and invasion (*Snail* and *Notch1*),^40^^,^^41^^,^^42^^,^^43^ resulting in the repression of tumor progression. In the context of GBC, only a few studies have reported the relationship between miR-34a-5p and GBC. For example, the low expression of miR-34a, which extends telomere length, is a useful biomarker for predicting poor prognosis in patients with GBC.^19^ In this study, we revealed that miR-34a-5p overexpression, as a therapeutic option, induced strong gene set enrichment of cell-cycle regulators with decreased expression levels related to cell proliferation, the epithelial-mesenchymal transition, and survival (e.g., *CDK6*, *BIRC5*, *Snail1*, and *Notch2*) in GBC cell lines and murine xenograft models. Thus, we strongly suggest that GBC cell growth inhibition by miR-34a-5p can aid in the development of therapeutic strategies for treating aggressive GBC.

Regarding the clinical application of miR-34a-5p supplementation, a liposomal miR-34a mimic, MRX34, has been developed, and the results of a Phase I study, in which patients with advanced solid tumors received MRX34 intravenously, were reported in 2020.^44^ Although the disease control rate was 29% in that study, the trial was closed early owing to four patient deaths with unexpected severe immune-mediated toxicities. Thus, specific drug delivery systems (DDSs) that avoid or minimize nonspecific delivery to other normal tissues require further elucidation.^45^ Alternatively, in current clinical practice, endoscopic ultrasonography-guided injections can be performed for GB lesions.^46^ Therefore, local miR-34a-5p supplementation, with or without systemic chemotherapy, may be a realistic option for clinicians to apply the results obtained in clinical settings.

In conclusion, although further optimization is required, our *in vitro* and *in vivo* analyses revealed that forced expression of miR-34a-5p presents a promising therapeutic option for patients with GBC.

## Materials and methods

### Organoid-based GB carcinogenesis model

Cre-transduced and pLKO.1-, a negative control construct, transduced GB organoids derived from *Kras*^LSL-G12D/+^; *Trp53*^f/f^ mice (a gift from Dr. Yoshitaka Hippo, Chiba Cancer Center Research Institute, Chiba, Japan). Detailed protocols for establishment and characterization have been described previously.^16^ Briefly, the GB was isolated from *Kras*^LSL-G12D/+^; *Trp53*^f/f^ mice, and normal GB organoids were established from these cells. Lentivirus-expressing Cre cells were transduced into GB organoids *in vitro* to establish GBC organoids. The tumorigenicity of the Cre-transduced GB organoids was confirmed by subcutaneously inoculating the organoids into nude mice, after which aggressively growing nodules were observed. Furthermore, the nodules were excised, and subsequent organoid cultures were established after the dissociation of the recovered tumor-derived epithelial cells. These tumor-derived organoids were used as Cre*-*transduced GB organoids to model GBC.^16^ Advanced DMEM/F12 (Thermo Fisher Scientific, Waltham, MA) media containing l-glutamine, penicillin, streptomycin, and amphotericin B, supplemented with 50 ng/mL epidermal growth factor (Peprotech, Rocky Hill, NJ), 100 ng/mL Noggin (Peprotech), 1 μM Jagged1 (AnaSpec, Fremont, CA), and 10 μM Y27632 (Wako, Osaka, Japan), were used with the Matrigel (no. 354234, Corning, Corning, NY) to culture organoids.

### Comprehensive miRNA expression analyses

For analysis, the Matrigel was lysed with Cell Recovery Solution (BD Biosciences, San Jose, CA) and washed with PBS to collect pure, viable organoid populations. Total RNA was extracted from these organoids using the 3D-Gene RNA Extraction Reagent (Toray Industries, Tokyo, Japan), and quality checked using an Agilent RNA 6000 Pico Kit and an Agilent 2100 Bioanalyzer (Agilent Technologies, Palo Alto, CA). Comprehensive miRNA expression analyses were performed using the 3D-Gene miRNA Labeling Kit and the 3D-Gene Mouse miRNA Oligo Chip (version 21; Toray Industries), as previously described.^47^ Fluorescent signals were scanned using a 3D-Gene Scanner 3000 and analyzed using 3D-Gene Extraction software (Toray Industries). The global normalization method was applied to background-subtracted signal intensities, setting the median of these signal intensities to 25.0. The FC values of Cre-transduced GB organoids for each miRNA were calculated using signals from pLKO.1*-*transduced GB organoids as reference.

### Cell culture and miRNA transfection

Human GBC cell lines G415, NOZ, and TGBC2TKB were obtained from Tohoku University (Sendai, Japan), the Japanese Collection of Research Bioresources cell bank (Osaka, Japan), and the RIKEN cell bank (Tsukuba, Japan), respectively. The NOZ cell line was established by Dr. S. Nagamori (National Institute of Infectious Diseases, Tokyo, Japan).^48^ G415 cells were cultured in RPMI 1640 (Thermo Fisher Scientific) supplemented with 10% fetal bovine serum (FBS), NOZ cells in Williams’ Medium E (Thermo Fisher Scientific) supplemented with 10% FBS and l-glutamine, and TGBC2TKB cells in DMEM (Thermo Fisher Scientific) containing low glucose and supplemented with 5% FBS. All of the cells were cultured in a humidified atmosphere containing 5% CO_2_ at 37°C.

Cells were seeded at 2–3 × 10^4^ cells/well in a 24-well plate, precultured in medium containing 5%–10% FBS up to 60% confluence, and then transfected with 3, 5, 10, or 20 nM of either mirVana miRNA mimic (hsa-miR-34a-5p [MC11030] or negative control no. 1 4464058) (Thermo Fisher Scientific) using Lipofectamine RNAiMAX Transfection Reagent (Invitrogen, Carlsbad, CA) according to the manufacturer’s protocol. The cells were cultured for 48–72 h and used for downstream assays.

### 2D cell culture viability and cell proliferation assays

Cell proliferation and viability were determined in 2D cells using an MTT (3-(4,5-dimethylthiazol-2-yl)-2,5-diphenyltetrazolium bromide) assay. Briefly, treated cells were seeded in 96-well plates at a density of 2 × 10^3^ or 2.5 × 10^3^ cells/well, depending on the cell line. After 24, 48, 72, and 96 h of incubation, MTT (0.5 mg/mL in the medium) was added to each well. The cells were then incubated for 3 h at 37°C, and the purple-blue formazan precipitate was dissolved using 200 μL DMSO. Absorbance was measured at 570 nm using a microplate reader (MULTISKAN GO, Thermo Fisher Scientific). Media containing the MTT reagent but without cells were used as a blank control. All of the experiments were performed in triplicate (minimum).

### Cell-cycle analysis

The treated cells were collected and fixed in 70% ethanol at 4°C for 2 h. The cells were then washed with PBS and stained with 20 μg/mL propidium iodide containing 0.25 mg/mL RNase for 30 min in a dark environment at 37°C. Finally, the cells in each cell-cycle phase were assayed using a MACS Quant Analyzer (Miltenyi Biotec, Bergisch Gladbach, Germany), and the percentage of cells in the G1, S, and G2/M phases was determined using FlowJo version 10 software (BD Biosciences, Ashland, OR).

### qRT-PCR

Total RNA was extracted from the organoids or cell lines using an miRNeasy Micro Kit (Qiagen, Valencia, CA) according to the manufacturer’s instructions. RNA samples were reverse transcribed using the TaqMan MicroRNA Reverse Transcription Kit (Applied Biosystems, Foster City, CA) with TaqMan MicroRNA Assay (Thermo Fisher Scientific). Subsequently, qPCR was performed using the TaqMan Fast Advanced Master Mix (Applied Biosystems) and the LightCycler 96 Real-Time PCR System (Roche, Basel, Switzerland) in 96-well plates. The analysis of relative gene expression data was calculated using the 2^−ΔΔCq^ method.^49^ All of the reactions were performed in duplicate.

The following TaqMan miRNA assays were performed: mmu (hsa)-miR-34a-5p (000426), mmu-miR-181a-5p (000480), mmu-miR-205-5p (000509), mmu-miR-378a-3p (002243), snoRNA202 (001232), and RNU6B (001093). Results were normalized to snoRNA202 and RNU6B expression levels in RNA samples derived from mice and *Homo sapiens*, respectively.

### Western blotting

After transfection with the miR-34a-5p mimic or negative control mimic for 48 or 72 h, protein lysates were harvested using radioimmunoprecipitation assay buffer (89900; Thermo Fisher Scientific) with protease inhibitors. The bicinchoninic acid method (23225; Thermo Fisher Scientific) was used to measure the protein concentration. Harvested cell protein (10 μg) was resolved using SDS-PAGE and transferred to polyvinylidene difluoride (PVDF) membranes (Bio-Rad, Hercules, CA) using semidry transfer. The membranes were blocked using a PVDF blocking reagent (Can Get Signal; Toyobo, Osaka, Japan) for 1 h. Reactive bands were detected using Clarity Western ECL Substrate (no. 1705060; Bio-Rad) and ImageQuant LAS 4000 (GE Healthcare Bio-Sciences AB, Uppsala, Sweden). The primary antibodies used in this study were against RasG12D (no. 14429, Cell Signaling Technology, Danvers, MA), p53 (no. 2524, Cell Signaling Technology), CDK6 (#3136, Cell Signaling Technology), cyclin D1 (no. 2922, Cell Signaling Technology), and β-actin (no. 4967, Cell Signaling Technology). Horseradish peroxidase-conjugated anti-mouse immunoglobulin G (IgG) (no. 7076, Cell Signaling Technology) or anti-rabbit IgG (no. 7074, Cell Signaling Technology) were used as secondary antibodies.

### Immunohistochemistry (IHC)

Organoid Matrigels were first depolymerized by Cell Recovery Solution and then embedded in iPGell (GenoStaff, Tokyo, Japan), followed by formalin fixation. Harvested subcutaneous tumor specimens were formalin fixed, paraffin embedded, and sectioned at 4 μm. H&E staining was used for histological analysis. For IHC analysis, tissue sections were deparaffinized and soaked in 0.3% H_2_O_2_ in methanol at room temperature for 10 min to block endogenous peroxidase activity. Antigen retrieval was performed by heating the specimens in 10 mM sodium citrate buffer (pH 6.0) using a microwave. After three 5-min washes with PBS, the tissue sections were incubated with a primary antibody against CDK6 (sc-7961; Santa Cruz Biotechnology, Dallas, TX) at room temperature for 30 min (1:200 dilution). After another three 5-min washes with PBS, the sections were incubated with secondary anti-mouse IgG (K4001; Agilent Technologies, Santa Clara, CA) for 30 min at room temperature. 3,3′-Diaminobenzidine+ (K3468, Agilent Technologies) was used as the chromogen, and the nuclei were counterstained with Mayer’s hematoxylin. CDK6 IHC slides were scanned using an Axio Scan.Z1 (Zeiss, Jena, Germany). The resulting whole-slide images were imported into an open-source software program (QuPath version 0.3.2) for viewing and assessment. Furthermore, a digital assistance tool was developed using QuPath’s positive cell detection algorithm and used for the assessment.^50^

### RNA-seq

Total RNA was isolated from NOZ cells treated with 5 nM miR-34a-5p mimic or negative control mimic 48 h after transfection. Three biological replicates were used for each sample (n = 3). The RNA quality was assessed using an Agilent 2100 Bioanalyzer (Agilent Technologies), and RNAs with an RNA integrity number above nine were processed for sequencing. Libraries were constructed using the NEBNext Ultra II Directional RNA Library Prep Kit (New England Biolabs, Ipswich, MA) according to the manufacturer’s instructions. Then, 150-bp paired-end sequencing was performed using an Illumina NovaSeq 6000 instrument (Illumina, San Diego, CA). Raw sequence data were filtered using Fastp (version 0.19.5) to remove adapter sequences and low-quality or short reads. The filtered data were aligned with the human reference genome (GRCh38.p13) using the STAR software (version 2.7.10a). Gene expression counts were summed using RSEM software (version 1.3.1).

The DEGs obtained from RNA-seq-based expression profiling were analyzed using the integrated Differential Expression and Pathway analysis online tools. DESeq2 results were used for differential expression analysis, and genes with an FDR < 0.01 and an absolute FC > 2 identified by DESeq2 were designated DEGs. The DEGs were then used to generate a heatmap after converting the data to base two logarithms and *Z* scores. The heatmap function of the ComplexHeatmap package (version 2.14) was used with R software (version 4.2.1; https://www.r-project.org/). The relationship between DEGs and miRNAs was assessed using miRTarBase,^51^ and enrichment analysis was conducted using ShinyGO 0.77.^52^ GSEA was performed using the clusterProfiler R package, KEGG pathway, and WikiPathway databases.^53^

### Dual luciferase reporter assay

Plasmids were constructed using the pmirGLO Dual-Luciferase miRNA Target Expression Vector (Promega, Madison, WI) for the binding site in the 3′ UTR of the potential target gene (*CDK6*) based on the miRNA target prediction database, miRDB.^54^ For the reported gene assay, NOZ cells (5 × 10^3^ cells) were cotransfected with reporter vectors *(CDK6* WT or *CDK6* MT), miR-34a-5p mimic, and negative control mimic using Lipofectamine 3000 Reagent (Invitrogen) on the Corning 96 Half Area Well Solid White Flat Bottom Polystyrene Tissue Culture-treated microplates (no. 3688; Corning). Following transfection for 72 h, luciferase activity was evaluated using the Nano-Glo Dual-Luciferase Reporter Gene Assay System (Promega) and GloMax Discover Microplate Reader (Promega). Normalization was performed using Renilla luciferase as the reference standard. All of the experiments were conducted in triplicate.

### *In vivo* experiments in mice bearing human tumor xenograft

For the *in vivo* model, NOZ cells (3 × 10^6^ cells) were subcutaneously injected into the flank regions of 6-week-old BALB/c nu/nu mice. Ten days later, local treatment with synthetic miRNAs prepared using atelocollagen as the DDS was initiated. Briefly, a mixture of miRNA-atelocollagen was prepared using either 1 nmol of the miR-34a-5p (HMI0508; Sigma-Aldrich, Saint Louis, MO) or 1 nmol of the negative control miRNA (HMC0003; Sigma-Aldrich) and an AteloGene Local Use “Quick Gelatin” kit (KOKEN, Tokyo, Japan), according to the manufacturer’s protocol.^55^^,^^56^ The mixture was injected around the tumor site five times over 3 weeks. The tumor volume was calculated using the following formula: V = A × B^2^/2 (mm^3^), where A is the largest diameter (mm) and B is the smallest diameter (mm). At 35 days after tumor cell implantation, the mice were euthanized, and the tumors were collected for further analysis.

The synthesized miRNA mimics used for *in vivo* experiments were as follows: hsa-miR-34a-5p mimic (sense, 5′-[AmC6]ACAACCAGCUAAGACACUGUCCA[dT][dT]-3′; antisense, 5′-UGGCAGUGUCUUAGCUGGUUGU-3′), and negative control miRNA (sense, 5′-[AmC6]GAUAUCCCGCCGCGAUCGUAUCCG[dT][dT]-3′; antisense, 5′-CGGUACGAUCGCGGCGGGAUAUC-3′). The animal protocol was approved by the Animal Care and Use Committee of Okayama University (approval no. OKU-2021537). All of the experiments were conducted in strict accordance with the Policy on the Care and Use of Laboratory Animals at Okayama University.

### Bioinformatics analyses

To investigate the miRNA expression in human tissue, we searched the GEO database for datasets using the keywords “gallbladder carcinoma” and “miRNA.” We used the GSE104165 dataset and the GEO2R online analysis tool to determine the expression levels of miRNAs of interest in GBC and normal tissues. The targets of these miRNAs were identified using the miRDB online database.^54^

### Statistical analyses

All of the statistical analyses were performed using JMP Pro 15.1.0 (SAS Institute, Cary, NC) or GraphPad Prism 9.3.1 (GraphPad, San Diego, CA). Group comparisons were performed using the Kruskal-Wallis, Mann-Whitney *U*, Pearson chi-square, or Wilcoxon rank-sum tests. All of the tests were two-sided. p < 0.05 were considered statistically significant.

## Data and code availability

All of the data, materials, and protocols used in this study are available from the corresponding author upon reasonable request.

## Supplemental information

Document S1. Figures S1‒S6

Document S2. Article plus supplemental information
