# Supplementary material for: MicroRNA-34a-5p: A pivotal therapeutic target in gallbladder cancer
Source: Mol Ther Oncol. 2024 Feb 8;32(1):200765. doi: 10.1016/j.omton.2024.200765 (PMC10963938; doi:10.1016/j.omton.2024.200765)
Supplement: Document S1. Figures S1‒S6 [file mmc1.pdf]

**OMTON, Volume *32***

## Supplemental information

# MicroRNA-34a-5p: A pivotal therapeutic target in gallbladder cancer

**Takashi Oda, Koichiro Tsutsumi, Taisuke Obata, Eijiro Ueta, Tatsuya Kikuchi, Soichiro Ako, Yuki Fujii, Tatsuhiro Yamazaki, Daisuke Uchida, Kazuyuki Matsumoto, Shigeru Horiguchi, Hironari Kato, Hiroyuki Okada, Ryota Chijimatsu, and Motoyuki Otsuka**

**Figure S1**

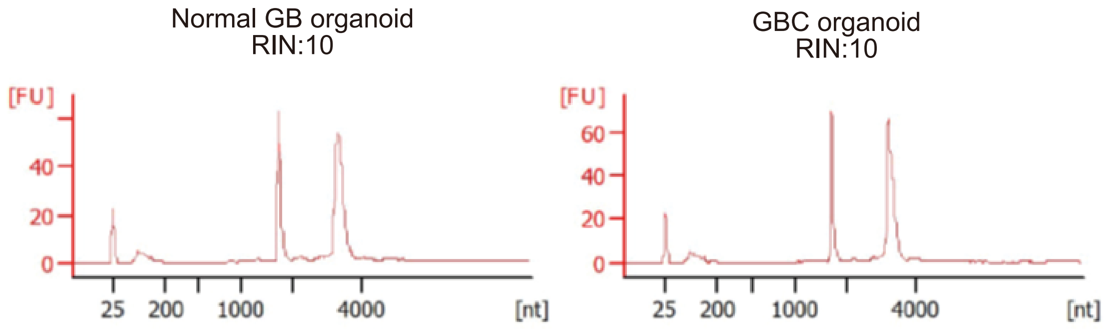


**Figure S1. Qualitative analyses of RNAs extracted from Gallbladder (GB) organoids for RNA-sequencing.**

Confirmation of high-quality RNA samples collected from Gallbladder cancer (GBC) organoids and normal GB organoids for RNA-sequencing sequencing Representative data from triplicate experiments are shown. RNA quality was confirmed using RNA integrity numbers (RIN).

## Figure S2

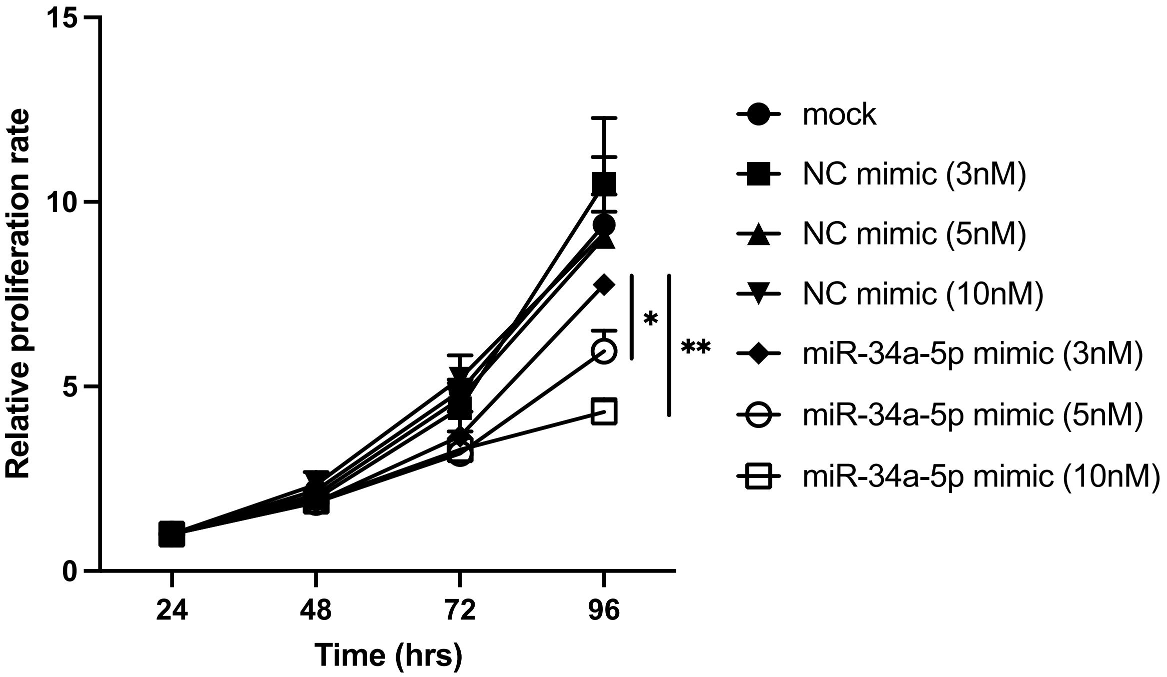


**Figure S2. Cell proliferation inhibition in a dose-dependent manner in NOZ cells transfected with miR-34a-5p mimic.**

Dose-dependent inhibition of NOZ cell proliferation by transfection with the miR-34a-5p mimic compared to the negative control, as determined by the MTT assay. Data are presented as the means ± SD (n = 3). *; P < 0.05. **; P < 0.01.

## Figure S3

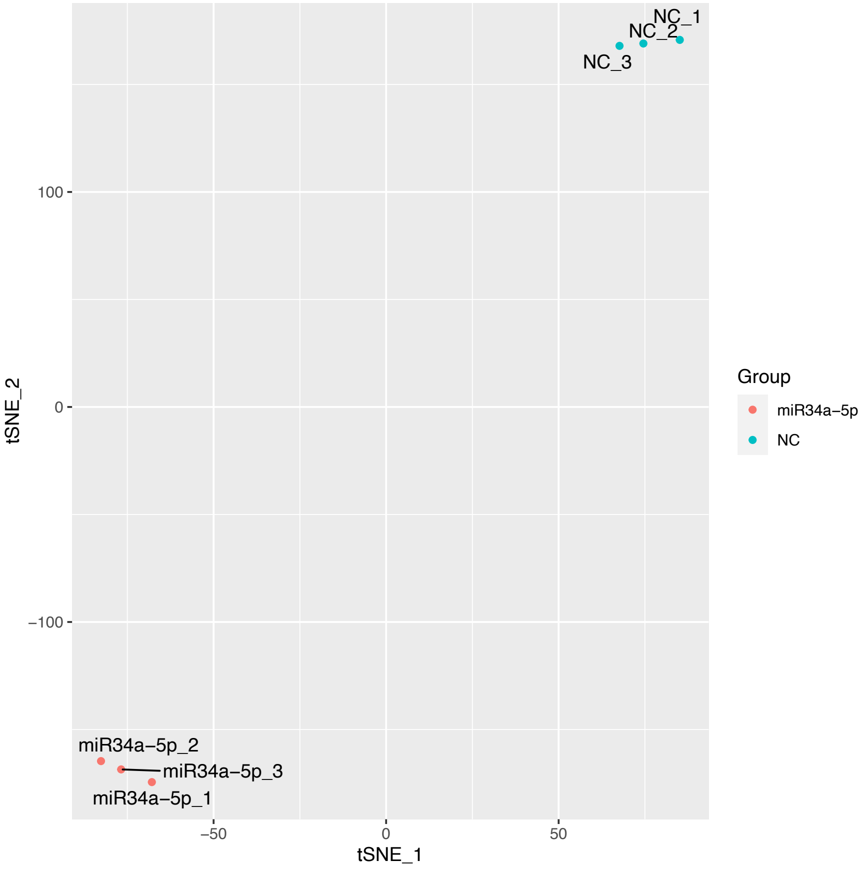


**Figure S3. Distinct clustering of the samples by the Principal Component Analysis (PCA) plot based on their gene expression patterns.**
The PCA plot based on the gene expression patterns showed distinct clustering between NOZ cells transfected with miR-34a-5p (n = 3) and those transfected with the negative control (n = 3).

## Figure S4

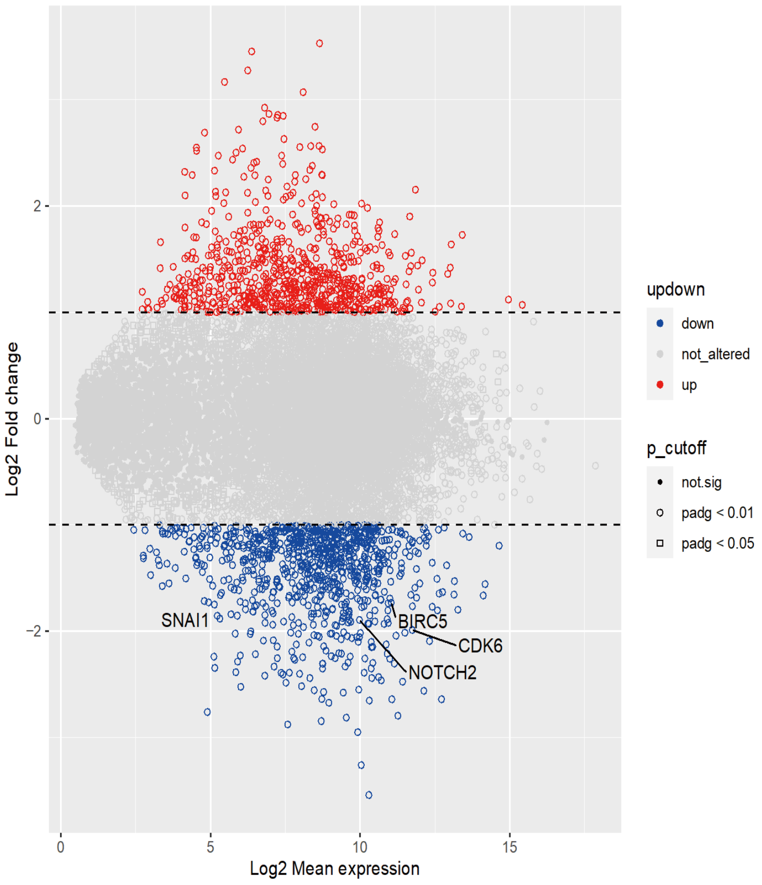


**Figure S4. miR-34a-5p target gene downregulation after forced miR-34a-5p expression.**
A volcano plot represents the differential gene expression status between NOZ cells transfected with miR-34a-5p mimics and controls. Representative miR-34a-5p target genes (*CDK6, BIRC5, Snail1,* and *Notch2*) are also shown.

# Figure S5

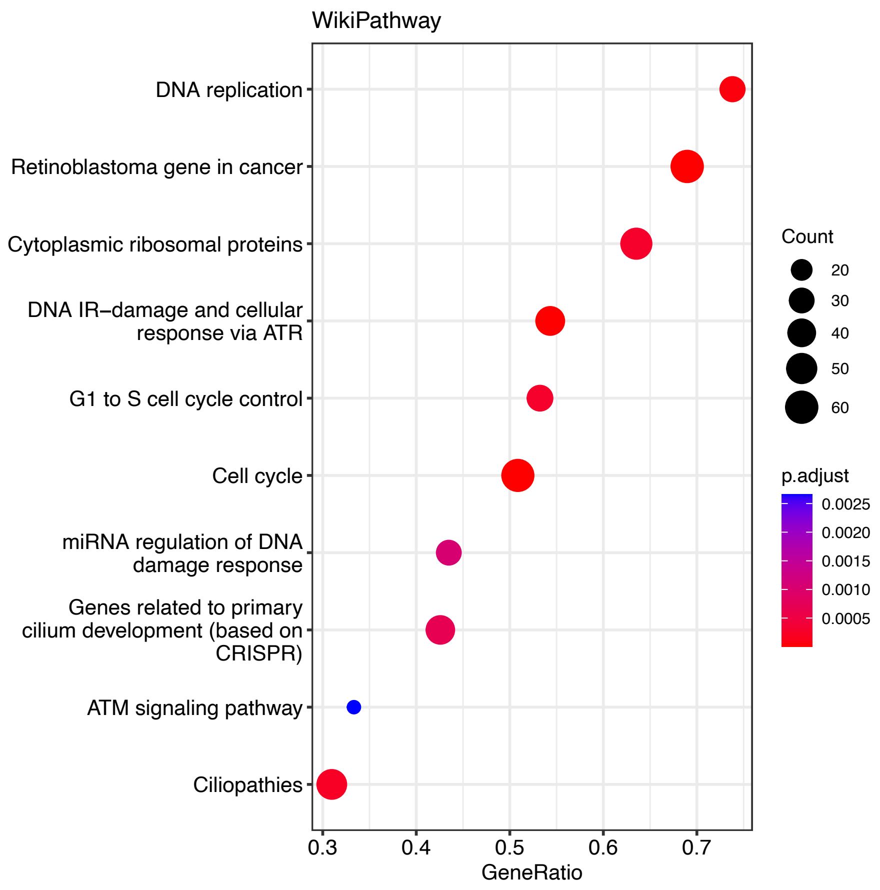


**Figure S5. Genes related to cell cycles are enriched after miR-34a-5p expression.**
Gene Set Enrichment Analysis using the Wikipathway shows the enrichment of the gene-sets related to “cell cycle” and “G1 to S cell cycle control” in NOZ cells transfected with miR-34a-5p compared to negative control (adjusted P = 4.11e-08 and 2.81e-04, respectively).

# Figure S6

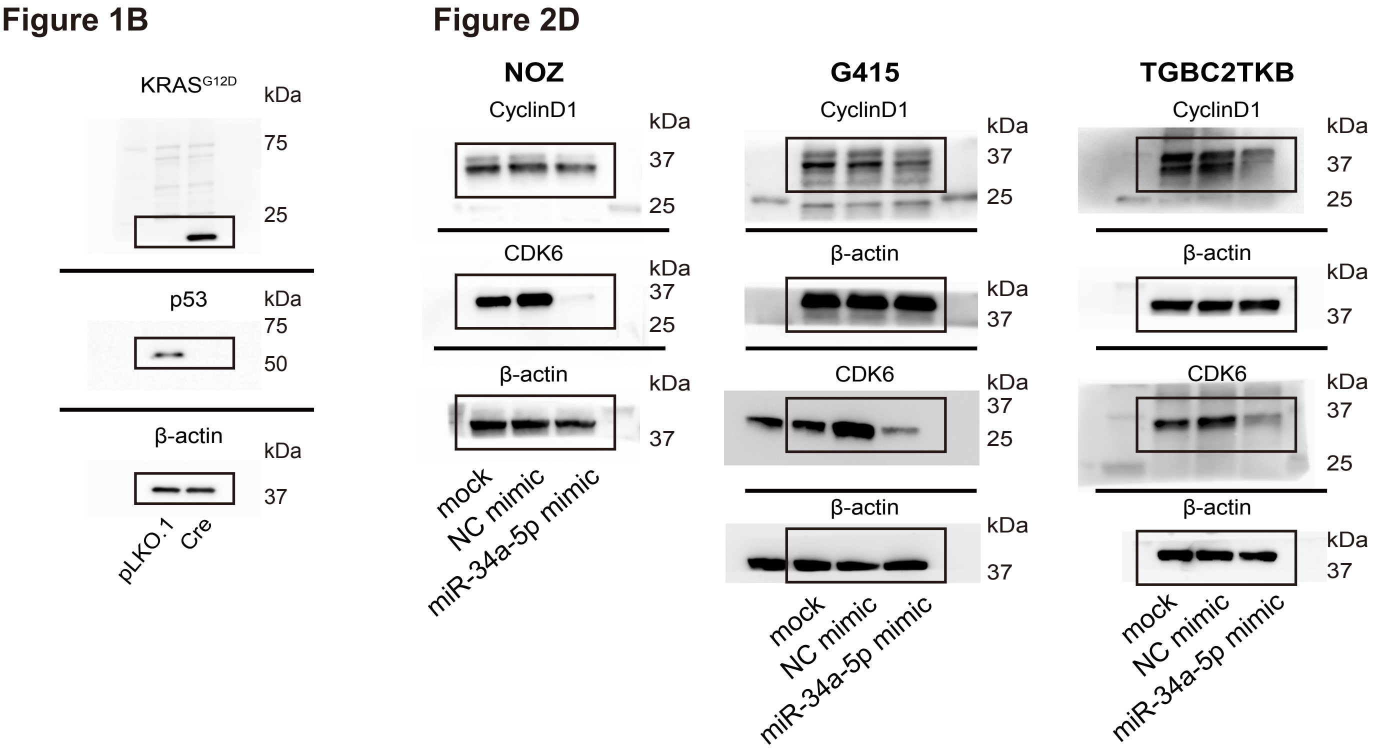


**Figure S6. Full blot images of Figure 1B and Figure 2D.**
